# Adaptive immune signature in HER2-positive breast cancer in NCCTG (Alliance) N9831 and NeoALTTO trials

**DOI:** 10.1038/s41523-022-00430-0

**Published:** 2022-05-24

**Authors:** Saranya Chumsri, Zhuo Li, Daniel J. Serie, Nadine Norton, Afshin Mashadi-Hossein, Kathleen Tenner, Heather Ann Brauer, Sarah Warren, Patrick Danaher, Gerardo Colon-Otero, Ann H. Partridge, Lisa A. Carey, Florentine Hilbers, Veerle Van Dooren, Eileen Holmes, Serena Di Cosimo, Olena Werner, Jens Bodo Huober, Amylou C. Dueck, Christos Sotiriou, Cristina Saura, Alvaro Moreno-Aspitia, Keith L. Knutson, Edith A. Perez, E. Aubrey Thompson

**Affiliations:** 1 Jacoby Center for Breast Health, Mayo Clinic, Jacksonville, FL USA; 2 Department of Health and Human Services, Mayo Clinic, Jacksonville, FL USA; 3 Department of Cancer Biology, Mayo Clinic, Jacksonville, FL USA; 4 NanoString, Inc., Seattle, WA USA; 5 Department of Health and Human Services, Mayo Clinic, Rochester, MN USA; 6 Dana-Farber Cancer Institute, Boston, MA USA; 7 The University of North Carolina, Chapel Hill, NC USA; 8 Breast International Group, Brussels, Belgium; 9 BrEAST Institut Jules Bordet, Brussels, Belgium; 10 The Frontier Science, Perth, UK; 11 Department of Applied Research and Technological Development, Fondazione IRCCS Istituto Nazionale dei Tumori, Milan, Italy; 12 Novartis, Basel-City, Switzerland; 13 Klinik für Frauenheilkunde und Geburtshilfe, Universitätsklinikum Ulm, Ulm, Germany; 14 Mayo Clinic, Scottsdale, AZ USA; 15 Institut Jules Bordet, Brussels, Belgium; 16 Vall d’Hebrón University Hospital, Vall d’Hebron Institute of Oncology (VHIO), SOLTI Breast Cancer Research Group, Barcelona, Spain; 17 Department of Immunology, Mayo Clinic, Jacksonville, FL USA

**Keywords:** Predictive markers, Breast cancer

## Abstract

Trastuzumab acts in part through the adaptive immune system. Previous studies showed that enrichment of immune-related gene expression was associated with improved outcomes in HER2-positive (HER2+) breast cancer. However, the role of the immune system in response to lapatinib is not fully understood. Gene expression analysis was performed in 1,268 samples from the North Central Cancer Treatment Group (NCCTG) N9831 and 244 samples from the NeoALTTO trial. In N9831, enrichment of CD45 and immune-subset signatures were significantly associated with improved outcomes. We identified a novel 17-gene adaptive immune signature (AIS), which was found to be significantly associated with improved RFS among patients who received adjuvant trastuzumab (HR 0.66, 95% CI 0.49–0.90, Cox regression model *p* = 0.01) but not in patients who received chemotherapy alone (HR 0.96, 95% CI 0.67–1.40, Cox regression model *p* = 0.97). This result was validated in NeoALTTO. Overall, AIS-low patients had a significantly lower pathologic complete response (pCR) rate compared with AIS-high patients (χ^2^ *p* < 0.0001). Among patients who received trastuzumab alone, pCR was observed in 41.7% of AIS-high patients compared with 9.8% in AIS-low patients (OR of 6.61, 95% CI 2.09–25.59, logistic regression model *p* = 0.003). More importantly, AIS-low patients had a higher pCR rate with an addition of lapatinib (51.1% vs. 9.8%, OR 9.65, 95% CI 3.24–36.09, logistic regression model *p* < 0.001). AIS-low patients had poor outcomes, despite receiving adjuvant trastuzumab. However, these patients appear to benefit from an addition of lapatinib. Further studies are needed to validate the significance of this signature to identify patients who are more likely to benefit from dual anti-HER2 therapy. ClinicalTrials.gov Identifiers: NCT00005970 (NCCTG N9831) and NCT00553358 (NeoALTTO).

## Introduction

Trastuzumab is a humanized IgG_1_ κ monoclonal antibody against the extracellular domain of human epidermal growth factor receptor 2 (HER2). There are several proposed mechanisms of action of trastuzumab, including inhibition of ligand-independent HER2 dimerization, inhibition of downstream signal transduction, induction of cell cycle arrest, induction of apoptosis, inhibition of angiogenesis, and DNA repair interference^1^. Besides these described mechanisms, multiple studies demonstrated that host immune response also plays a pivotal role in the antitumor effects of trastuzumab^1–3^. In contrast, lapatinib is a dual-receptor tyrosine kinase inhibitor against HER2 and epidermal growth factor receptor (EGFR). Lapatinib primarily acts through inhibition of HER2 signaling cascade^4^. The effects and interactions between lapatinib and the immune system remain largely unknown.

More recently, several studies have demonstrated the prognostic implications for pre-existing immune response in various cancers, including breast cancer. One method to evaluate pre-existing immune activation is to assess the amount of tumor-infiltrating lymphocytes (TILs) in baseline tumor samples prior to treatment. While the prognostic value of TILs is more established in triple-negative breast cancer (TNBC), its significance in HER2-positive breast cancer is still somewhat controversial. In TNBC, multiple studies have consistently shown that higher TILs are associated with improved pathological response to neoadjuvant chemotherapy as well as long-term outcome^5,6^. In contrast, previous studies in HER2-positive breast cancer have shown conflicting results. TILs have been shown to be associated with improved outcomes in HER2-positive breast cancer patients treated with adjuvant chemotherapy in the BIG 02–98 trial^7^ as well as an abbreviated course of trastuzumab and adjuvant chemotherapy in the FinHER trial^8^. However, with longer exposure to trastuzumab, TILs were not associated with outcome in patients who received trastuzumab-based adjuvant chemotherapy in the North Central Cancer Treatment Group (NCCTG, now Alliance for Clinical Trials in Oncology) N9831 study^9^. However, most studies only evaluated TILs in the stroma and lack global assessment of the immune landscape of the whole tissue section as intratumoral TILs are more challenging to assess on hematoxylin and eosin (H&E) slides^10^. Furthermore, the scoring of TILs is somewhat subjective and can be operator-dependent. Currently, there is no consensus for a clinically relevant TIL threshold or cutoff^10^. Besides these technical challenges, the nature and phenotype of these TILs cannot be assessed solely based on morphological appearance. In this study, we sought to analyze molecular signatures of different subsets of tumor-infiltrating immune cell populations using gene expression data to assess different immune subtypes in correlation with outcome in patients who were treated with trastuzumab-based adjuvant chemotherapy in the N9831 as well as trastuzumab and/or lapatinib-based chemotherapy in the NeoALTTO trials.

## Results

### Patient characteristics in the N9831 and NeoALTTO trials

Patient characteristics in the N9831 and NeoALTTO trials are described in Table 1. From the total of 3505 patients enrolled in the N9831 trial, 1268 patients had complete gene expression data and were included in this analysis. The median follow-up in this analysis is 10.6 years (range 0.8–15.3 years), and the median age was 50.0 (22.0–80.0) years. Overall, 670 (52.8%) patients had estrogen-receptor (ER) and/or progesterone-receptor (PR)-positive disease and 598 (47.2%) patients had ER/PR-negative disease. There were 445 (35.1%) patients in arm A, 449 (35.4%) patients in arm B, and 374 (29.5%) patients in arm C. In the NeoALTTO trial, a total of 455 patients were enrolled. Of those, 244 patients had complete RNA-seq data and were included for analysis. The median follow-up was 6.6 years (range 0.2–8.1 years), and the median age was 49.0 years (range 23.0–79.0 years). In this trial, 119 (48.8%) patients had estrogen-receptor (ER)-positive disease, and 125 (51.2%) patients had ER-/PR-negative disease. There were 77 (31.6%) patients in arm 1 with trastuzumab alone, 85 (34.8%) patients in arm 2 with lapatinib alone, and 82 (33.6%) patients in arm 3 with the combination.

**Table 1:** Patient characteristics in the NCCTG N9831 and NeoALTTO.

	N9831 (*N* = 1268)	NeoALTTO (*N* = 244)	Total (*N* = 1512)	*P* value^a^
*Age* (years)				0.1968
Mean (SD)	50.0 (±10.6)	48.9 (±11.3)	49.9 (±10.7)	
Median	50.0	49.0	50.0	
Range	(22.0–80.0)	(23.0–79.0)	(22.0–80.0)	
*Tumor size* (cm)				<0.0001
Mean (SD)	2.9 (±1.8)	10.8 (±15.4)	4.2 (±7.0)	
Median	2.5	4.2	2.6	
Range	(0.1–15.0)	(2.1–90.0)	(0.1–90.0)	
*Lymph node status*				<0.0001
N0 or N1	745 (58.8%)	206 (84.4%)	951 (62.9%)	
N2 or N3	523 (41.2%)	38 (15.6%)	561 (37.1%)	
*Tumor grade*				<0.0001
Missing	15	122	137	
Grade 1	23 (1.8%)	11 (9.0%)	34 (2.5%)	
Grade 2	324 (25.9%)	57 (46.7%)	381 (27.7%)	
Grade 3	906 (72.3%)	54 (44.3%)	960 (69.8%)	
*ER status*				0.2440
Negative	598 (47.2%)	125 (51.2%)	723 (47.8%)	
Positive	670 (52.8%)	119 (48.8%)	789 (52.2%)	
*ARM*^b^				0.3635
A or 1	445 (35.1%)	85 (34.8%)	530 (35.1%)	
B or 2	449 (35.4%)	77 (31.6%)	526 (34.8%)	
C or 3	374 (29.5%)	82 (33.6%)	456 (30.2%)	

^a^Wilcoxon rank-sum test was used for continuous variables and Chi-square test was used for categorical variables.

^b^Arm A, B, and C in NCCTG N9831 and Arm 1, 2, and 3 in NeoALTTO trial.

### CD45, immune-subset signatures, and outcome with trastuzumab-based therapy in N9831

**Fig. 1:**
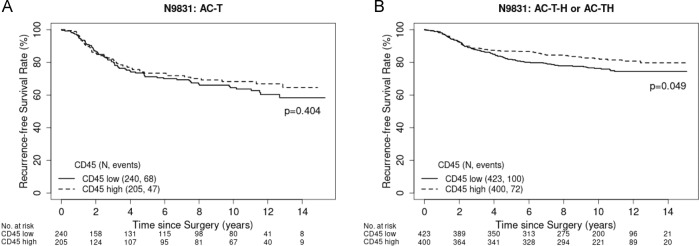
Kaplan–Meier curves of RFS in the NCCTG N9831 comparing between patients with CD45 high vs. low. **A** Chemotherapy-only arm (AC–T). **B** Sequential (AC–T–H) or concurrent trastuzumab arm (AC–TH).

In all treatment arms combined, CD45 as a categorical variable (high vs. low) in multivariable analysis was significantly associated with improved RFS (HR 0.75, 95% CI 0.59–0.95, *p* = 0.02) after adjusting for tumor size, lymph node status, tumor grade, age, and ER/PR status. However, CD45 was not associated with improved RFS in patients who received chemotherapy alone in arm A (HR 0.87, 95% CI 0.60–1.28, Cox regression model *p* = 0.48), but was significantly associated with improved outcomes in patients who received trastuzumab-based adjuvant chemotherapy in arms B and C (HR 0.72, 95% CI 0.53–0.97, Cox regression model *p* = 0.03). Similar findings were observed with Kaplan–Meier estimates. In all treatment arms combined, the 14-year Kaplan–Meier estimates for RFS were 69.32% (95% CI 65.28–73.61%) in the CD45-low group and 75.47% (95% CI 71.29–79.89%, log-rank *p* = 0.03) in the CD45-high group. Among patients who received chemotherapy alone, there was no significant difference in the 14-year RFS with 58.32% (95% CI 50.45–67.43%) in the CD45-low group and 64.62% (95% CI 56.28–74.19%) in the CD45-high group (log-rank *p* = 0.40). However, there was a significant improvement in 14-year RFS among patients who received trastuzumab plus chemotherapy in arms B and C with 74.4% (95% CI 70.07–79%) in the CD45-low group vs. 79.7% (95% CI 75.22–84.44%, log-rank *p* = 0.049) in the CD45-high group (Fig. 1).

**Fig. 2:**
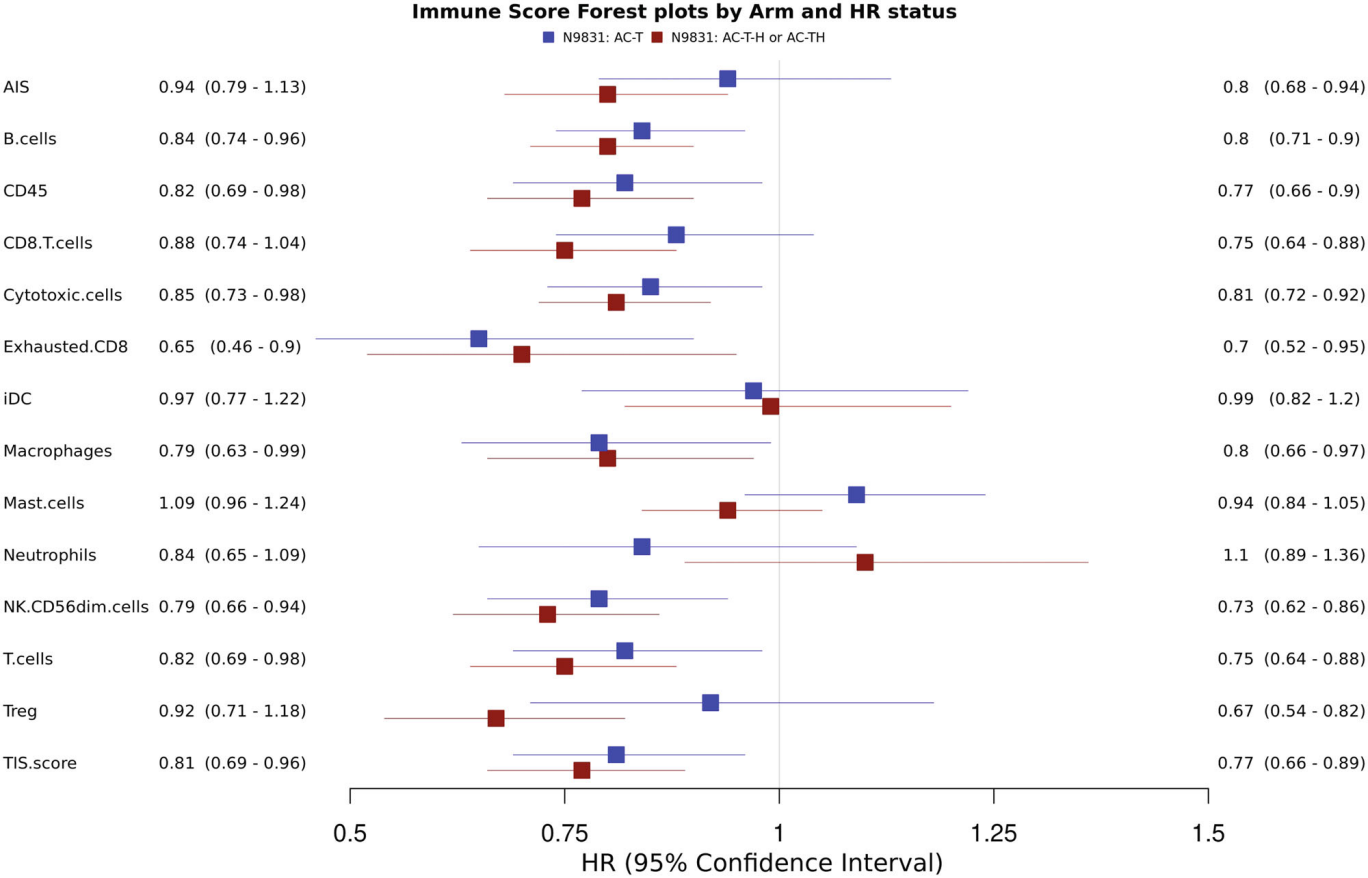
Forest plot of RFS with immune-subset signatures in the NCCTG N9831 with chemotherapy-only arm (AC–T) in blue and sequential (AC–T–H) or concurrent trastuzumab arm (AC–TH) in red. The HR on the forest plots was from multivariate models adjusting for age, nodal status, ER/PR status, tumor size, and tumor grade. The size of the box in the forest plots represents the precision (standard error) of the estimates.

In addition to CD45, 13 additional immune-subset signatures were evaluated in both treatment arms (Fig. 2). For patients who received chemotherapy alone, gene signatures related to B cells, cytotoxic cells, exhausted CD8, macrophages, NK CD56dim, T cells, and tumor inflammation signature (TIS) score were significantly associated with improved RFS. Among patients who received trastuzumab-based adjuvant chemotherapy, a similar pattern was observed with gene signatures related to B cells, cytotoxic cells, exhausted CD8, NK CD56dim, T cells, and TIS score being significantly associated with a better outcome. However, gene signatures related to CD8 T cells and Tregs were only associated with significantly improved outcomes in trastuzumab-based adjuvant chemotherapy arms.

### Adaptive immune signature and outcome with trastuzumab-based therapy in N9831

**Fig. 3:**
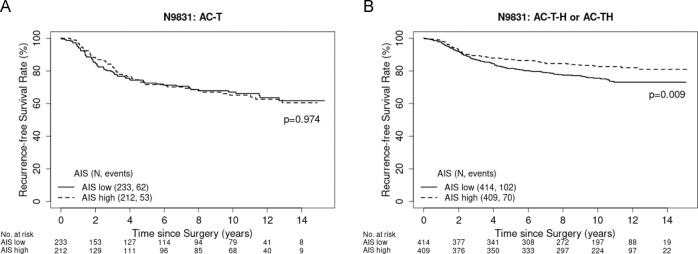
Kaplan–Meier curves of RFS in the NCCTG N9831 comparing between AIS-high vs. -low patients. **A** Chemotherapy-only arm (AC–T). **B** Sequential (AC–T–H) or concurrent trastuzumab arm (AC–TH).

Further evaluation of the contribution from each immune-related gene and outcome in patients treated with trastuzumab-based adjuvant chemotherapy was carried out. The Cox proportional hazards regression model was used to assess genes significantly associated with the improved outcome with *p* < 0.05. Subsequently, Gene Ontology and Gene Set Enrichment Analysis were used to identify pathways or biological processes correlated with these genes. Among these biological processes, we identified the “adaptive immune reactome” as one of the top biological processes associated with improved outcomes. Based on the genes listed in this reactome, we developed an adaptive immune signature (AIS). Enrichment of this 17-gene AIS (Supplementary Table 1) was found to be significantly associated with improved RFS in the overall population (HR 0.76, 95% CI 0.60–0.96, Cox regression model *p* = 0.02). Similar to CD45, AIS is only significantly associated with improved outcomes in patients who received trastuzumab-based adjuvant chemotherapy (HR 0.66, 95% CI 0.49–0.90, Cox regression model *p* = 0.01), but not in patients who received chemotherapy alone (HR 0.96, 95% CI 0.67–1.40, Cox regression model *p* = 0.97, Fig. 3). Compared with the AIS-low group, AIS-high patients were significantly older (Wilcoxon rank-sum test, *p* = 0.01), had smaller tumors (Wilcoxon rank-sum test, *p* = 0.0007), and had ER-negative tumors (χ^2^ *p* = 0.02), but there was no difference in lymph node status or tumor grade (Table 2).

**Table 2:** Patient characteristics among AIS-low group vs. AIS-high group in the NCCTG-N9831 trial.

	AIS-low (*N* = 647)	AIS-high (*N* = 621)	Total (*N* = 1268)	*P* value^a^
*Age* (years)				0.0099
Mean (±SD)	49.3 (±10.6)	50.8 (±10.6)	50.0 (±10.6)	
Median	49.0	51.0	50.0	
Range	(22.0–80.0)	(23.0–77.0)	(22.0–80.0)	
*Tumor size* (cm)				0.0007
Mean (±SD)	3.0 (±1.9)	2.7 (±1.6)	2.9 (±1.8)	
Median	2.5	2.4	2.5	
Range	(0.1–13.2)	(0.1–15.0)	(0.1–15.0)	
*Nodal status*				0.7410
N0	120 (18.5%)	126 (20.3%)	246 (19.4%)	
N1	261 (40.3%)	238 (38.3%)	499 (39.4%)	
N2	181 (28.0%)	168 (27.1%)	349 (27.5%)	
N3	85 (13.1%)	89 (14.3%)	174 (13.7%)	
*Menopausal status*				0.1845
1	345 (53.3%)	308 (49.6%)	653 (51.5%)	
2	302 (46.7%)	313 (50.4%)	615 (48.5%)	
*Tumor grade*				0.1374
Missing	7	8	15	
Grade 2	189 (29.5%)	158 (25.8%)	347 (27.7%)	
Grade 3	451 (70.5%)	455 (74.2%)	906 (72.3%)	
*ER/PR status*				0.0235
Negative	285 (44.0%)	313 (50.4%)	598 (47.2%)	
Positive	362 (56.0%)	308 (49.6%)	670 (52.8%)	
*ARM*				0.7571
A	233 (36.0%)	212 (34.1%)	445 (35.1%)	
B	224 (34.6%)	225 (36.2%)	449 (35.4%)	
C	190 (29.4%)	184 (29.6%)	374 (29.5%)	

^a^Wilcoxon rank-sum test was used for continuous variables and Chi-square test was used for categorical variables.

### CD45, immune-subset signatures, adaptive immune signature, and outcome with lapatinib-based therapy in NeoALTTO

Overall, in patients from all treatment arms combined in the NeoALTTO trial, none of the immune-subset signatures were significantly associated with pathological response (Supplement Table 2). However, when stratified by the treatment arm, 6 immune-subset signatures were significantly associated with pCR but only in the trastuzumab-alone arm. Similar to the N9831 trial, CD45 was associated with the improved outcomes with a higher pCR rate among patients treated with trastuzumab-based therapy in the NeoALTTO trial. Other immune-subset signatures significantly associated with a higher pCR rate included B cells, exhausted CD8, macrophage, NK CD56dim cells, and TIS (Supplementary Fig. 2).

**Fig. 4:**
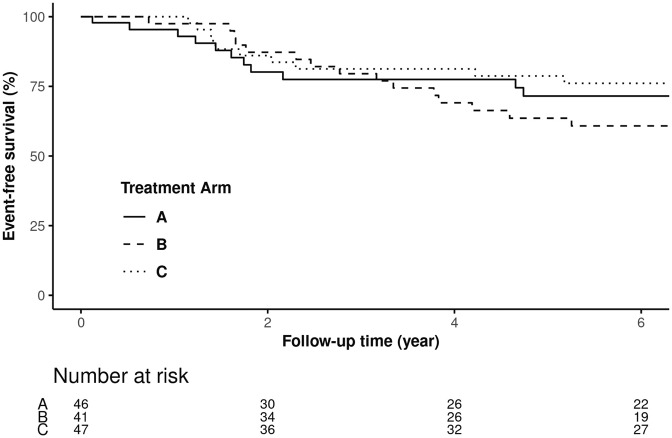
Kaplan–Meier curves of AIS-low patients in the NeoALTTO. Patients in arm A were treated with lapatinib alone, arm B with trastuzumab alone, and arm C with the combination of trastuzumab and lapatinib.

For the AIS, patients with AIS-high tumors had a significantly higher pCR rate compared with patients with AIS-low tumors in the trastuzumab-alone arm (41.7% vs. 9.8%). Using univariable logistic regression analysis, this difference was significant with OR of 6.61 (95% CI 2.09–25.59, logistic regression model *p* = 0.003). However, there was no significant difference in pCR rate among AIS-high vs. AIS-low patients treated in lapatinib-alone arm or the combination arm (Table 3). More notably, among AIS-low patients, which accounted for 54.9% (*n* = 134 out of 244) of patients, the pCR rate is significantly higher in patients who received the combination of trastuzumab and lapatinib compared with trastuzumab single agent (51.1% vs. 9.8%, OR 9.65, 95% CI 3.24–36.09, logistic regression model *p* < 0.001). A similar trend was observed with 6-year EFS. Among AIS-low patients, 6-year EFS rate was numerically higher in those treated with lapatinib (71.5%, 95% CI 58.5–87.5%) or the combination of trastuzumab and lapatinib (76.1%, 95% CI 64.2–90.24%), compared with trastuzumab alone (60.8%, 95% CI 47.1–78.5%, Fig. 4). Using univariable Cox regression analysis, the difference among patients treated with trastuzumab had HR of 0.59 (95% CI 0.25–1.39), but was not statistically significant (Cox regression model *p* = 0.226) likely may be due to the small sample size.

**Table 3:** Complete pathological response rate (%pCR) with univariable logistic regression analysis predicting for pCR and %6-year event-free survival (EFS) rate in the NeoALTTO trial with univariable Cox regression analysis predicting for EFS based on treatment arms and adaptive immune-signature status (high vs. low).

Treatment arms	%pCR AIS low (*N* = 134)	%pCR AIS high (*N* = 110)	OR^a^ (95%CI, *p* value)	%6-yr EFS AIS low (95%CI)	%6-yr EFS AIS high (95%CI)	HR^b^ (95%CI, *p* value)
Arm 1: lapatinib	15.2% (7)	23.1% (9)	1.67 (0.56–5.17, 0.358)	71.5% (58.5–87.5%)	62.3% (47.9–81.1%)	1.35 (0.61–2.98, 0.457)
Arm 2: trastuzumab	9.8% (4)	41.7% (15)	6.61 (2.09–25.59, 0.003)	60.8% (47.1–78.5%)	76.2% (63.1–92.1%)	0.59 (0.25–1.39, 0.226)
Arm 3: combination of trastuzumab and lapatinib	51.1% (24)	45.7% (16)	0.81 (0.33–1.94, 0.632)	76.1% (64.2–90.24%)	81.1% (68.5–96.01%)	0.85 (0.32–2.24, 0.741)

^a^The outcome was modeled with pCR using the Logistic regression model.

^b^The outcome was modeled with the composite endpoint of progression, relapse, or death using the Cox regression model.

## Discussion

Several studies demonstrated that trastuzumab exerts its activity via both innate and adaptive immune systems. One of the proposed immune-based mechanisms of trastuzumab is antibody-dependent cellular cytotoxicity (ADCC)^2^. ADCC is the process that involves lysing of antibody-bound target cells by immune effector cells through the interaction between fragment-crystallizable (Fc) region of the immunoglobulin and Fc gamma-receptors (FcγR) on immune effector cells. These effector cells include a variety of immune cells, including NK cells, neutrophils, and γδT cells^11^. The binding of the immunoglobulin to FcγR on antigen-presenting cells can also facilitate Fcγ-mediated phagocytosis of the immune complex and enhance antigen presentation as well as tumor-specific T-cell activation. Multiple studies showed that pre-existing host immune response measured in the forms of TILs or immune-related gene signatures is associated with the improved outcomes with trastuzumab^12^. Consistent with these previous studies, our study also demonstrated that global immune infiltration measured by CD45 and other immune-subset signatures is associated with improved outcomes in patients who received trastuzumab-based adjuvant chemotherapy. CD45 or leukocyte common antigen is a glycoprotein expressed in all nucleated hematopoietic cells, except for erythrocytes and platelets^13^. In our study, a higher level of CD45 mRNA expression in the entire tumor section was associated with a 25% improvement in RFS in all treatment arms combined in the N9831 trial. Among patients who receive trastuzumab, there was a 28% improvement in outcome. While there was no significant difference in outcome among patients treated with chemotherapy only, the total number of patients in arm A alone was small. Furthermore, the confidence intervals were overlapping and, thus, the significant difference observed may also possibly be due to sample size. Nevertheless, this result is in contrast to the previous study from the same N9831 trial in which stromal TILs were not associated with improved outcomes in patients who received trastuzumab-based adjuvant chemotherapy^9^. However, only TILs in the stroma were assessed in that particular study. In addition, in that same trial, gene expression and network analyses also showed that higher expression ≥9 out of 14 immune-function genes was associated with improved outcomes in patients treated with trastuzumab^14^. Besides global immune infiltrate with CD45, other specific immune-subset signatures that confer better outcomes with trastuzumab include B cells, cytotoxic cells, exhausted CD8, NK CD56dim, T cells, and TIS score. Using gene enrichment analysis, we also identified a novel AIS that encompasses 17 genes related to adaptive immune response. However, immune-related genes in this AIS do not overlap with previously reported 14 immune-function genes in the N9831 using cDNA-mediated Annealing, Selection, Extension, and Ligation or DASL assay^14^. The lack of concordance may have to do with the methods used to measure mRNA abundance, DASL microarray vs. NanoString. Enrichment of AIS was associated with improved outcomes only among patients treated with trastuzumab, but not in patients who received chemotherapy alone. Among patients who received trastuzumab, there was a 34% improvement in RFS observed in AIS-high patients.

Nevertheless, the significance of pre-existing host immune response and outcome in patients treated with a small-molecule inhibitor such as lapatinib is less known. Moreover, there is currently no specific predictive immune biomarker to determine the benefit of the combination of trastuzumab and lapatinib over single-agent trastuzumab to date. In the previous neoadjuvant trastuzumab and lapatinib trials, several studies demonstrated that enrichment of immune-related gene signatures was associated with increased pCR. These clinical trials include CALGB 40601^15^, CherLOB^16^, GeparSixto^17^ and NeoALTTO^18^ trials. However, these immune-related gene signatures are associated with increased pCR as a whole rather than in a specific treatment arm. In another publication by Fumagalli et al.^18^ from the NeoALTTO Trial, ERBB2, PAM50 HER2-high subtype, ESR1, and Genomic Grade Index were significantly associated with increased pCR. In addition, two immune gene signatures were significantly associated with higher pCR but only in the combination arm. However, this significant interaction test was only observed when comparing between the combination arm and combined trastuzumab and lapatinib arms, but was no longer significant when compared with the single-agent trastuzumab arm. Nevertheless, the loss of significance may be due to the smaller sample size when treatment arms were separated.

In our study, enrichment of AIS in the NeoALTTO trial was associated with higher pCR when all treatment arms were combined. Consistent with our previous findings from the N9831 trial, AIS-high patients had improved outcomes and higher pCR with single-agent trastuzumab. The pCR rate in this group of patients was 48% compared with 8% in AIS-low patients who received trastuzumab (OR 9.12, 95% CI 2.25–54.42, *p* = 0.0004). More importantly, AIS-low patients appeared to have improved outcomes when trastuzumab was given in combination with lapatinib. In this group of patients, the pCR rate was 8% with single-agent trastuzumab, but increased to 52% with an addition of lapatinib (OR 13.0, 95% CI 3.4–75.4, *p* < 0.0001). Therefore, AIS appeared to provide not only prognostic value for HER2-positive breast cancer patients receiving trastuzumab-based therapy, but also provided predictive value for dual anti-HER2 therapy with a small-molecule inhibitor like lapatinib. Given that the AIS-low phenotype is associated with younger age, larger tumor size, and ER-positive disease, these groups of patients may benefit from dual anti-HER2 therapy with a small-molecule inhibitor. Besides lapatinib, there are two additional small-molecule inhibitors approved for HER2-positive breast cancer, namely neratinib and tucatinib. Among these three small-molecule inhibitors, neratinib is currently the only agent approved in patients with early-stage HER2-positive breast cancer after completion of trastuzumab-based adjuvant chemotherapy. The approval was based on the ExteNET trial^19,20^, which was a randomized multicenter phase-III trial of neratinib vs. placebo after completing a year of maintenance trastuzumab. Consistent with our findings, subset analysis in the ExteNET trial demonstrated more pronounced benefit of neratinib in the hormone-receptor-positive subgroup (HR 0.51, 95% CI 0.33–0.77) compared with the hormone-receptor-negative subgroup (HR 0.93, 95% CI 0.60–1.43)^19^. However, given the difference in the methods to determine gene expression with N9831 using NanoString and NeoALTTO using RNA-seq, it is not possible to identify an appropriate cutoff for AIS in our current study. Therefore, additional studies will be needed to define clinically applicable cutoff points for future clinical use.

In summary, our study demonstrated the potential predictive value of AIS to identify which patients are more likely to benefit from dual anti-HER2 therapy with trastuzumab in combination with a small- molecule inhibitor lapatinib. However, further studies are needed to validate AIS in a larger set of samples, as well as to evaluate the potential cutoff point and predictive value of AIS with other anti-HER2 small-molecule inhibitors.

## Methods

### Patient population

N9831 trial^21^ was a randomized phase-III trial that enrolled patients with high-risk node-negative or node-positive HER2-positive breast cancer in an adjuvant setting. Patients were randomized to 3 treatment arms, including arm A treated with chemotherapy alone with doxorubicin and cyclophosphamide followed by paclitaxel (AC–T), arm B treated with similar chemotherapy followed by sequential trastuzumab (AC–T–H), and arm C treated with trastuzumab concurrently with weekly paclitaxel (AC–TH). Recurrence-free survival (RFS), defined as the time from random assignment to breast cancer recurrence (local, regional, or distant recurrence of breast cancer) or breast cancer-related death, was available in the N9831.

NeoALTTO trial^22^ was used as our validation cohort. This trial was a randomized, open-label, phase-III neoadjuvant trial of chemotherapy in combination with trastuzumab (arm 1), lapatinib (arm 2), or the combination of trastuzumab and lapatinib (arm 3). In this trial, operable HER2-positive breast cancer patients were randomized to receive trastuzumab, lapatinib, or the combination for 6 weeks. After that, the assigned anti-HER2 therapies were continued in combination with weekly paclitaxel for 12 weeks, followed by surgery. After surgery, additional chemotherapy with three cycles of fluorouracil, epirubicin, and cyclophosphamide was given. Event-free survival (EFS), which was defined as the time from randomization to disease progression during neoadjuvant chemotherapy, post-surgery breast cancer relapse, and second primary malignancy or death without recurrence, was available in the NeoALTTO trial. Trial schemas are depicted in Supplementary Fig. 1.

Participants in both trials signed an IRB-approved, protocol-specific informed consent document in accordance with the Declaration of Helsinki, International Ethical Guidelines for Biomedical Research Involving Human Subjects (CIOMS), Belmont Report, and U.S. Common Rule.

### CD45 and immune-subset signatures

In the N9831 trial, NanoString^TM^ technology was used to quantify mRNA in paraffin-embedded tumor samples using a specifically designed custom code set at Dr. Thompson’s laboratory (Mayo Clinic, Jacksonville, FL) as described in previous publications^23,24^. NanoString custom code sets were constructed to comprise 1252 genes, including five housekeeping genes (*B2M, GAPDH, POLR2A, UBC*, and *YWHAZ*) for normalization purposes. Immune-subset signatures were calculated using normalized and log2-transformed data based on the previous publication by Danaher et al.^25^. The geometric mean across relevant genes was calculated to generate the composite score for each immune-subset signature. CD45 was used to assess global leukocyte infiltration. For other immune- subset signatures, groups of genes as described in Supplementary Table 1 were used to determine B cells, CD8 T cells, cytotoxic cells (e.g., NK and CD8 T cells), exhausted CD8, immature dendritic cells (iDC), macrophages, mast cells, neutrophils, NK CD56dim, T cells, and regulatory T cells (Treg). A similar algorithm was used to calculate the adaptive immune-signature (AIS) score using 17 genes listed in Supplement Table 1. AIS score was calculated in the following steps. First, conditional quantile normalization was used to normalize the expression. This type of normalization normalizes library size, gene length, GC content, and output-normalized values in the log2 scale. Second, for each patient, sum the cqn-normalized values of these 17 genes and divide by 17. Third, standardization was performed, such that the mean expression is 0 and the variance is 1. The score was mean-centered and standardized in all analyses.

In the NeoALTTO trial, baseline-biopsied samples prior to treatment were used for RNA sequencing. The NEB Next Ultra directional RNA Library Preparation Kit was used to construct strand-specific complementary DNA libraries. Sequencing was performed on the HiSeq 2500 system (Illumina) per the standard operating procedure of GATC Biotech AG. RNA-sequencing data processing, including reading-pair trimming and alignment, was described in the previous publication^18^. Existing data from this publication^18^ were used for the analysis.

### Statistical analysis

Statistical analysis was based on the data lock from November 14, 2017, in the NCCTG-N9831 trial and October 9, 2017, in the NeoALTTO trial. Patient characteristics were summarized as median with range and mean with standard deviation. RFS at different time points was estimated, and survival curves were drawn using the Kaplan–Meier method. The Cox regression model was used to evaluate the impact of CD45, immune-subset signatures, and AIS enrichment on RFS one at a time to assess their individual impacts with and without adjustment of patient characteristics. Cox proportional hazard models were used to generate point estimates of hazard ratios (HRs) and corresponding 95% confidence intervals (CIs) to assess the benefit of trastuzumab for RFS, EFS, and overall survival (OS). Forest plots were used to visualize the hazard ratios for each immune score. Odds ratio (OR) with 95% CIs was calculated for pathological complete response (pCR) in each treatment arm. High vs. low gene and gene signatures were determined using the median as the cutoff for all gene signatures and CD45. All tests were two-sided with an alpha level set at 0.05 for statistical significance. Enrichment analysis with Gene Ontology and Gene Set Enrichment Analysis was performed on genes with significant HR to identify potential associated biological processes and generate a signature associated with outcome.

### Reporting summary

Further information on research design is available in the Nature Research Reporting Summary linked to this article.

## Supplementary information

Supplementary

Reporting Summary checklist

## Data Availability

Due to the informed consent form, data privacy, and Intellectual Property Rights related restrictions, the clinical data cannot be made public, i.e., accessible for anyone, for any purpose without a review process and without putting an agreement in place. Nevertheless, raw data are available upon request, and any requests can be directed to the NCCTG-N9831 and central (Neo)ALTTO teams. The full trial protocols are available in the Protocol Exchange.
