# Supplementary material for: Adaptive immune signature in HER2-positive breast cancer in NCCTG (Alliance) N9831 and NeoALTTO trials
Source: NPJ Breast Cancer. 2022 May 24;8:68. doi: 10.1038/s41523-022-00430-0 (PMC9130150; doi:10.1038/s41523-022-00430-0)

Supplementary

Supplementary Table 1: Gene list for immune subset signatures

Immune Subset Signatures	Gene List
B cells	MS4A1, TNFRSF17, KIAA0125
CD8 T cells	CD8A, FLT3LG
Cytotoxic cells	GNLY, GZMA, GZMB, KLRB1, KLRK1, NKG7
Exhausted CD8	LAG3, PTGER4
Immature dendritic cells	CARD9, F13A1
Macrophages	CD163, CD68, CD84, MS4A4A
Mast cells	TPSAB1, CPA3, TPSB2
Neutrophils	CSF3R, FCGR3B
NK CD56 dim	IL21R
T cells	CD3D, CD3E, CD3G, CD6, TRAT1
Regulatory T cells	FOPXP3
TIS	CCL5, CD27, CD274, CD276, CD8A, CMKLR1, CXCL9, CXCR6, HLA-DQA1, HLA-DRB1, HLA-E, IDO1, LAG3, NKG7, PDCD1LG2, PSMB10, STAT1, TIGIT
AIS	CD200R1, CD226, TRAF6, CTLA4, CD3G, ICOS, ITGAL, MAP3K8, CD28, NFKBIA, ICAM3, CSK, SELL, PDPK1, C3, CDH1, HRAS

Supplementary Table 2: Immune subset signatures and pathological response including no complete pathological response (non-pCR) vs. complete pathological response (pCR) in patients from all treatment arms combined in the NeoALTTO trial.

	Non-pCR (N=169)	pCR (N=75)	Total (N=244)	P value
B cells				0.5436
Mean	0.0	0.0	0.0	
Median	0.0	0.1	0.0	
Range	(-2.7-2.6)	(-3.3-2.2)	(-3.3-2.6)	
CD45				0.7628
Mean	0.0	0.0	0.0	
Median	-0.1	0.2	-0.1	
Range	(-2.6-2.6)	(-3.0-2.7)	(-3.0-2.7)	
CD8 T cells				0.4755
Mean	0.0	0.0	0.0	
Median	-0.2	0.1	-0.1	
Range	(-3.3-2.2)	(-2.4-2.1)	(-3.3-2.2)	
Cytotoxic cells				0.2735
Mean	0.0	0.1	0.0	
Median	-0.1	0.2	0.0	
Range	(-2.6-2.2)	(-3.1-2.4)	(-3.1-2.4)	
Exhausted CD8				0.0804
Mean	-0.1	0.2	0.0	
Median	0.0	0.1	0.0	
Range	(-2.6-2.7)	(-2.5-2.2)	(-2.6-2.7)	
iDC				0.7095
Mean	0.0	0.0	0.0	
Median	0.1	0.1	0.1	
Range	(-2.4-3.5)	(-2.6-2.6)	(-2.6-3.5)	

	Non-pCR (N=169)	pCR (N=75)	Total (N=244)	p value
Macrophages				0.6913
Mean	0.0	0.0	0.0	
Median	0.0	0.0	0.0	
Range	(-2.4-3.0)	(-2.3-2.2)	(-2.4-3.0)	
Mast cells				0.5255
Mean	0.0	0.0	0.0	
Median	0.1	0.0	0.1	
Range	(-3.3-2.5)	(-3.1-1.7)	(-3.3-2.5)	
Neutrophils				0.9914
Mean	0.0	0.0	0.0	
Median	0.1	0.1	0.1	
Range	(-3.6-2.1)	(-2.0-2.6)	(-3.6-2.6)	
NK CD56^{dim} cells				0.4634
Mean	0.0	0.1	0.0	
Median	0.0	0.0	0.0	
Range	(-2.8-2.4)	(-1.7-2.2)	(-2.8-2.4)	
T cells				0.4035
Mean	0.0	0.0	0.0	
Median	-0.2	0.2	-0.1	
Range	(-2.7-2.2)	(-3.0-2.3)	(-3.0-2.3)	
Treg				0.6462
Mean	0.0	0.1	0.0	
Median	0.0	0.2	0.1	
Range	(-4.2-2.1)	(-4.2-4.2)	(-4.2-4.2)	
TIS				0.3659
Mean	0.0	0.1	0.0	
Median	0.0	0.2	0.1	
Range	(-2.2-2.4)	(-2.8-2.1)	(-2.8-2.4)	

Supplementary Figure 1: The NCCTG N9831 and NeoALTTO trial schemas.

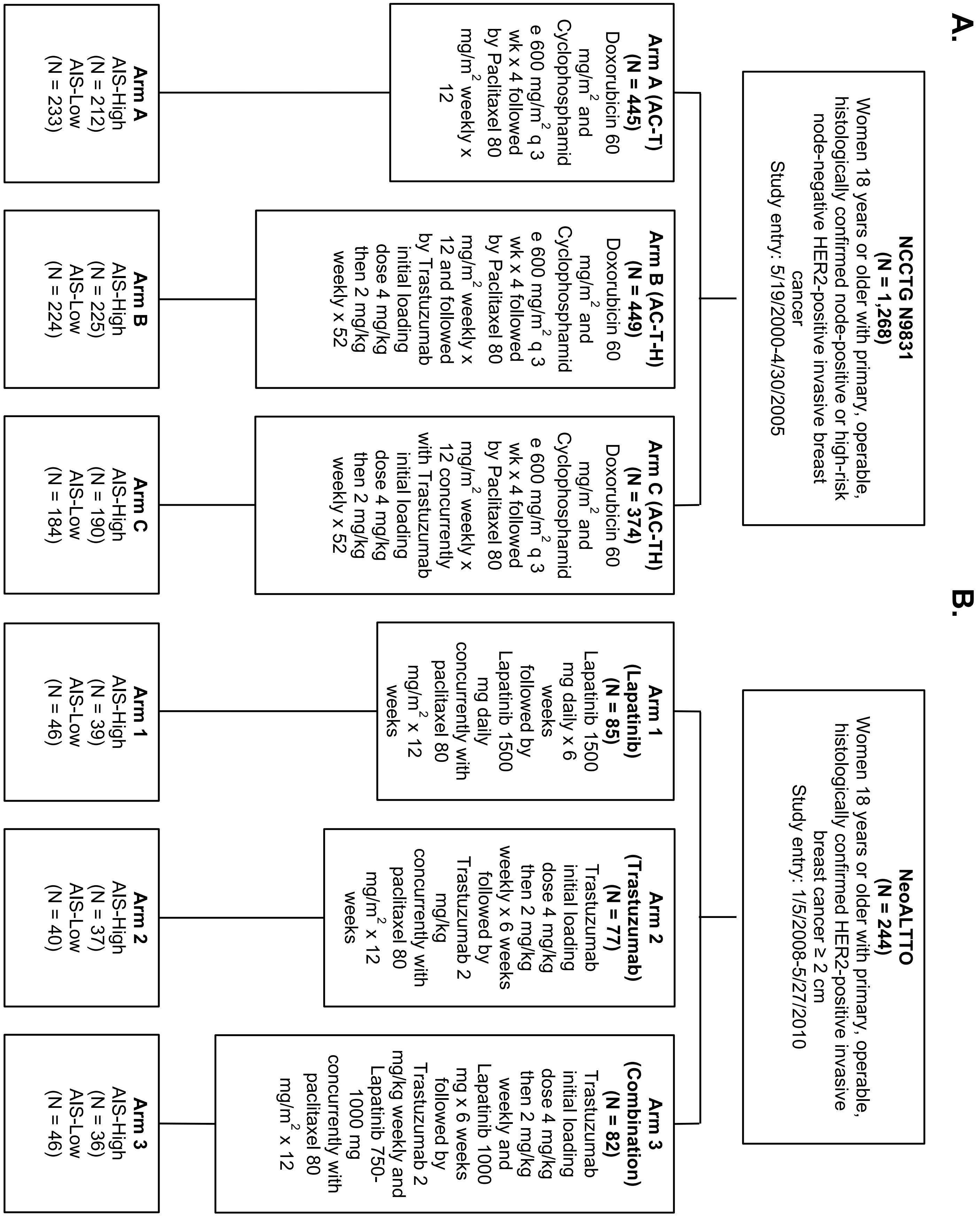

Supplementary Figure 2: Immune subset signatures significantly associated with higher pCR only in trastuzumab alone arm in the NeoALTTO trial.

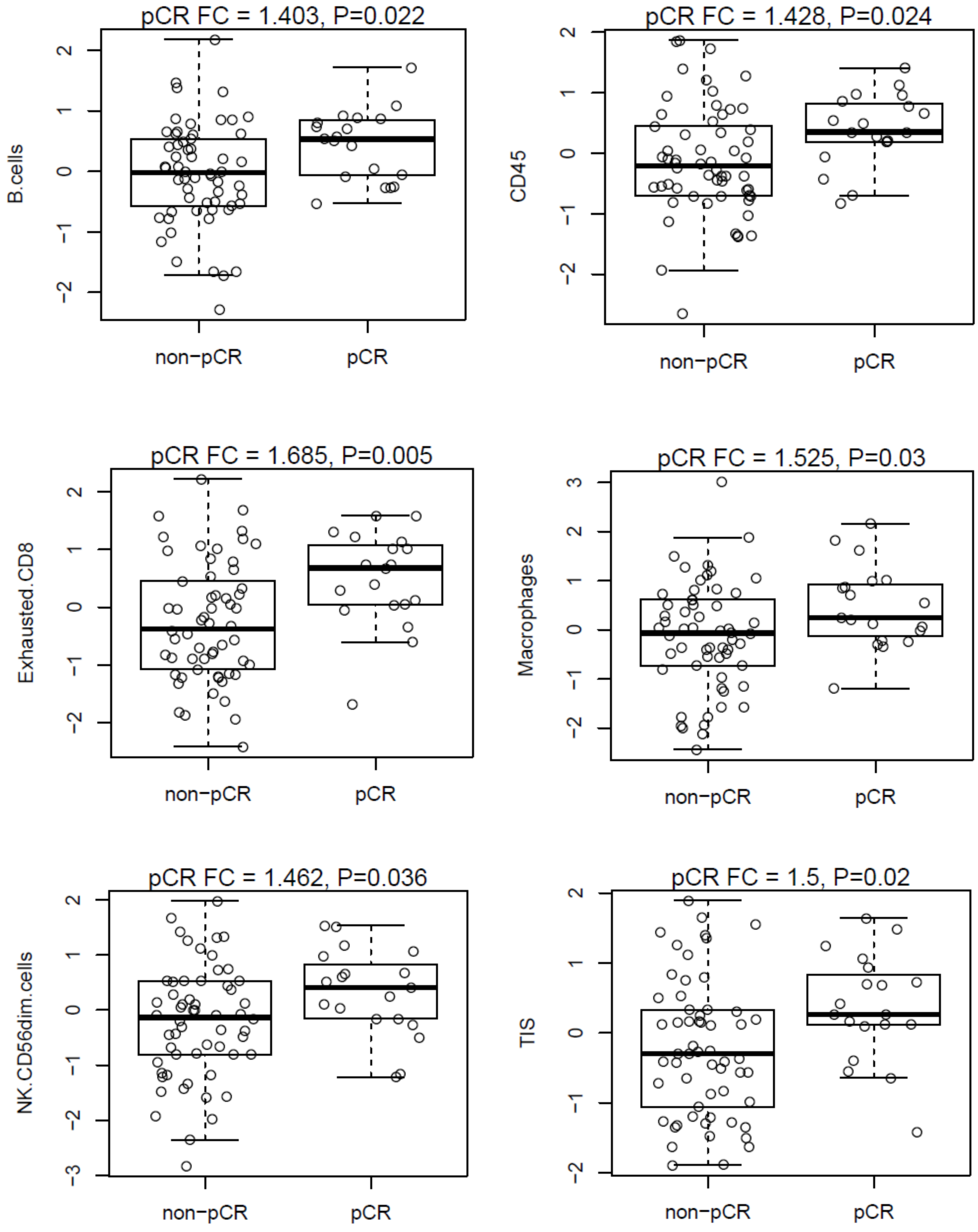

Supplement: Supplementary file 1 — Supplementary [file 41523_2022_430_MOESM1_ESM.pdf]
